# SV2B defines a subpopulation of synaptic vesicles

**DOI:** 10.1093/jmcb/mjad054

**Published:** 2023-09-08

**Authors:** Isabelle Paulussen, Hannes Beckert, Timothy F Musial, Lena J Gschossmann, Julia Wolf, Mathieu Schmitt, Jérôme Clasadonte, Georges Mairet-Coello, Christian Wolff, Susanne Schoch, Dirk Dietrich

**Affiliations:** Synaptic Neuroscience Team, Department of Neurosurgery, University Hospital Bonn, Bonn 53127, Germany; Synaptic Neuroscience Team, Department of Neuropathology, University Hospital Bonn, Bonn 53127, Germany; Microscopy Core Facility, Medical Faculty, University of Bonn, Bonn 53127, Germany; Microscopy Core Facility, Medical Faculty, University of Bonn, Bonn 53127, Germany; Synaptic Neuroscience Team, Department of Neurosurgery, University Hospital Bonn, Bonn 53127, Germany; Synaptic Neuroscience Team, Department of Neuropathology, University Hospital Bonn, Bonn 53127, Germany; Synaptic Neuroscience Team, Department of Neurosurgery, University Hospital Bonn, Bonn 53127, Germany; Synaptic Neuroscience Team, Department of Neuropathology, University Hospital Bonn, Bonn 53127, Germany; UCB Pharma, Braine l'Alleud 1420, Belgium; UCB Pharma, Braine l'Alleud 1420, Belgium; UCB Pharma, Braine l'Alleud 1420, Belgium; UCB Pharma, Braine l'Alleud 1420, Belgium; Synaptic Neuroscience Team, Department of Neuropathology, University Hospital Bonn, Bonn 53127, Germany; Synaptic Neuroscience Team, Department of Neurosurgery, University Hospital Bonn, Bonn 53127, Germany

**Keywords:** 3D electron microscopy, synaptic vesicles, synaptic vesicle proteins, endocytosis, trafficking, spatial organization

## Abstract

Synaptic vesicles can undergo several modes of exocytosis, endocytosis, and trafficking within individual synapses, and their fates may be linked to different vesicular protein compositions. Here, we mapped the intrasynaptic distribution of the synaptic vesicle proteins SV2B and SV2A in glutamatergic synapses of the hippocampus using three-dimensional electron microscopy. SV2B was almost completely absent from docked vesicles and a distinct cluster of vesicles found near the active zone. In contrast, SV2A was found in all domains of the synapse and was slightly enriched near the active zone. SV2B and SV2A were found on the membrane in the peri-active zone, suggesting the recycling from both clusters of vesicles. SV2B knockout mice displayed an increased seizure induction threshold only in a model employing high-frequency stimulation. Our data show that glutamatergic synapses generate molecularly distinct populations of synaptic vesicles and are able to maintain them at steep spatial gradients. The almost complete absence of SV2B from vesicles at the active zone of wildtype mice may explain why SV2A has been found more important for vesicle release.

## Introduction

Structurally, the most remarkable feature of a neuronal synapse and the most detectable feature in electron micrographs is the prominent accumulation of synaptic vesicles. Small excitatory cortical synapses typically contain at least 50–100 vesicles (Harris and Sultan, 1995; Schikorski and Stevens, 1997; Zhu et al., 2021), and their aggregation fills and likely shapes the synapse. Individual synaptic vesicles (∼50 nm wide) look very similar and are structurally indistinguishable. Nevertheless, two distinct populations of vesicles can be defined based on their location within the synapse, namely docked and non-docked synaptic vesicles. The small region of the presynaptic membrane delimiting the synaptic cleft (∼200 nm × 200 nm) is called the active zone (AZ), which is composed of a set of enriched, specialized fusion proteins and calcium channels to allow for calcium-triggered, rapid synaptic vesicle release (Südhof, 2012). Only a handful of vesicles are found in contact with the membrane at the AZ and thus are termed ‘docked’. Studies correlating structure to function have consistently provided evidence that morphologically docked vesicles correspond to the so-called readily releasable vesicles, which are ready to undergo fusion in response to a single action potential (Rosenmund and Stevens, 1996; Murthy et al., 2001; Schikorski and Stevens, 2001). All other vesicles in a synapse are non-docked and provide a large reservoir of vesicles that are more or less remote from the AZ, hereafter referred to as vesicles in the remote domain.

To allow for repeated releases of neurotransmitters, synapses have developed effective mechanisms to rapidly replenish docked vesicles after their release. This process involves endocytosis, re-filling, and re-docking of exocytosed vesicles. Endocytosis likely happens near the AZ and can be remarkably quick (<1 sec) or happens on a slower timescale (tens of seconds) (Soykan et al., 2016; Tehran and Maritzen, 2022). Depending on the level of activity, retrieved vesicles are preferentially used to replenish docked vesicles or integrated into vesicles in the remote domain (Rey et al., 2015). Synaptic vesicles from everywhere in the synapse can principally move to and undergo release at the AZ (Park et al., 2012). Synaptic vesicle release happens not only in response to action potentials but also spontaneously in the absence of neuronal firing. Spontaneous release may preferentially occur at the periphery of the AZ and involve the slightly different release machinery (Kavalali, 2015; Chanaday and Kavalali, 2018; Wang et al., 2022) or even different types of vesicles (Ramirez et al., 2012). Vesicles released spontaneously or by action potentials are mobile within synapses (Peng et al., 2012). Taken together, synaptic vesicles can undergo multiple fates during the cycle of exocytosis and endocytosis (Chanaday et al., 2019), probably linked to different vesicle localizations in the synapse. Vesicles at all positions are in mutual exchange during different levels of activity, which raises the question of how trafficking and positioning of vesicles are coordinated.

At present, the factors determining where a vesicle is routed within the synapse and under which conditions it is released and endocytosed are not well understood, but it seems that the membrane proteins of synaptic vesicles play a key role. There are at least 40 (or more) different resident proteins in each synaptic vesicle. For several of these proteins, such as synaptotagmin, synaptobrevin, synaptophysin, VGlut, and ATPase, specific roles in the life cycle of a vesicle have been defined (Rizo, 2022). It has been suggested that while vesicles are morphologically indistinguishable, they may be differentially equipped with sets of proteins that are crucial for vesicle fate in the synapse (Chanaday and Kavalali, 2018; Chanaday et al., 2019). For instance, overexpressing or knocking down the non-canonical soluble *N*-ethylmaleimide-sensitive-factor attachment receptor (SNARE) VAMP4/7 or Vti1a (Chanaday and Kavalali, 2018; Lin et al., 2020), respectively, preferentially affects some modes of release over others. However, as these manipulations may not exclusively affect a specific subpopulation of vesicles, it is difficult to determine whether these results point to a specific function of the SNAREs or the existence of differentially equipped vesicles within a synapse.

To date, mapping of individual vesicular proteins throughout entire synapses has not been performed. Therefore, it remains unknown whether discrete vesicle identities can be maintained during recycling and whether subpopulations of synaptic vesicles may assume preferred positions within a synapse. If unique vesicle identities exist, neurons could regulate different modes of release and endocytosis by manufacturing the required type of vesicle.

Synaptic vesicle protein 2 (SV2) is a ubiquitous membrane glycoprotein consistently found on all vesicles in synapses, with very little variability between vesicles (Mutch et al., 2011; Ciruelas et al., 2019; Stout et al., 2019). The SV2 family of membrane glycoproteins consists of three members, SV2A, SV2B, and SV2C, and all of them have been linked to multiple neurological disorders, e.g. epilepsy, Parkinson's disease, Alzheimer's disease, and cognitive disorders (Stout et al., 2019). Although the exact functional role of SV2 family members has not been elucidated, it was established that they are important for efficient evoked release and appear to facilitate the progression of docked synaptic vesicles to a release-competent state (Crowder et al., 1999; Janz et al., 1999; Xu and Bajjalieh, 2001; Custer et al., 2006; Chang and Südhof, 2009; Ciruelas et al., 2019).

Most glutamatergic synapses express both SV2A and SV2B, two major SV2 paralogues. At the cellular level, synaptic deficits after deletion of SV2A can be rescued by expression of SV2B, suggesting some degree of functional redundance (Ciruelas et al., 2019). In light of this equivalence and their widespread co-expression in glutamatergic neurons, it is unexpected that only ablation of SV2A but not SV2B causes a pronounced reduction in synaptic glutamate release (Custer et al., 2006). This dominant role of SV2A raises the possibility that SV2A and SV2B may segregate into different vesicle populations within a synapse that in turn might be differentially recruited for synaptic release. Here, we mapped SV2B and SV2A throughout glutamatergic synapses in the CA1 region of the mouse hippocampus via electron microscopy (EM) and showed strikingly different intrasynaptic distributions of these two paralogues.

## Results

### Verification of pre-embedding immunogold staining of SV2B

**Figure 1:**
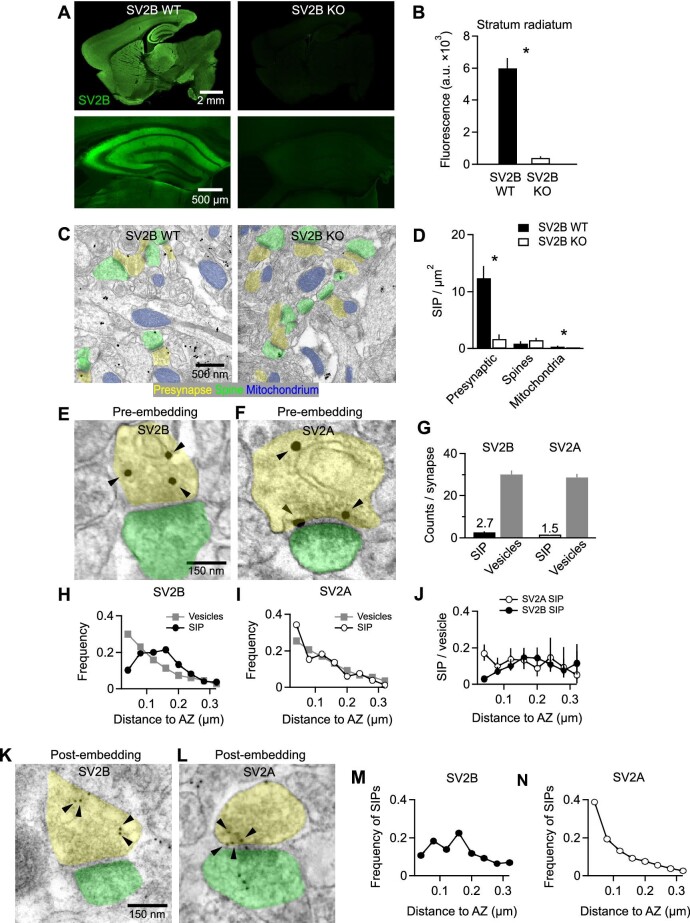
SV2B is not close to the synaptic cleft. (**A**) Exemplary images of immunohistochemical staining of SV2B in SV2B wildtype (WT) and SV2B knockout (KO) mouse brain sections (upper row: whole brain; lower row: hippocampus). SV2B KO images are almost completely devoid of any fluorescent signal, indicating the specificity of the anti-SV2B antibody. (**B**) Quantitative analysis of SV2B mean fluorescence intensity in the stratum radiatum of the hippocampal CA1 region in SV2B wildtype and knockout mice. *n *= 4 mice per group. (**C**) Exemplary STEM images of immunogold labeling of SV2B in brain sections from SV2B wildtype and knockout mice. Note the reduced number of SIPs in SV2B knockout mice. (**D**) In SV2B wildtype brain sections, SV2B SIPs are enriched in presynaptic boutons (presynaptic) and almost completely absent from spines and mitochondria. In comparison, SV2B SIP density is significantly reduced in SV2B knockout presynaptic boutons and mitochondria. *n *= 5 mice per group. (**E** and **F**) Exemplary STEM images of pre-embedding immunogold labeling of SV2B and SV2A in wildtype brain sections. Note that SV2A but not SV2B is found close to the AZ. Arrowheads mark SIPs. Color code: yellow, presynaptic boutons; green, spines. (**G**) The number of SIPs or vesicles observed per synapse positive for SV2B or SV2A. Note that the lower number likely reflects a typical low labeling efficacy (see Discussion for details). (**H** and **I**) The frequencies of SV2B (**H**) or SV2A (**I**) SIPs and synaptic vesicles at different distances to the AZ. SV2B SIPs are rarely found close to the AZ, while SV2A SIPs and synaptic vesicles exhibit a similar distribution, peaking in proximity to the AZ. A total of 75 SV2B-positive synapses (including 201 SIPs and 2189 vesicles) and 100 SV2A-positive synapses (including 154 SIPs and 2739 vesicles) were analyzed, respectively. *n *= 5 mice per group. (**J**) The frequency of SV2A or SV2B SIPs per synaptic vesicle per distance bin (corrected for the difference in labeling efficacy between SV2A and SV2B). Data are shown as the ratio per bin ± 95% confidence interval of the ratio calculated across all synapses. SV2B and SV2A labeling probabilities on vesicles near the AZ are 0.03 and 0.17 with 95% confidence intervals (0.02, 0.05) and (0.13, 0.22), respectively. A total of 75 SV2B-positive synapses and 100 SV2A-positive synapses were analyzed, respectively. *n *= 5 mice per group. (**K** and **L**) Exemplary STEM images of post-embedding immunogold labeling of SV2B and SV2A in wildtype brain sections. Arrowheads mark SIPs. (**M** or **N**) The frequency of SV2B (**M**) or SV2A (**N**) SIPs at different distances to the AZ in scans obtained after post-embedding immunogold labeling. SV2B is rarely found in proximity to the AZ. Note that the counting of vesicles was not possible as the ultrastructure was not sufficiently preserved in the majority of the samples. A total of 94 SV2B-positive synapses and 108 SV2A-positive synapses were analyzed, respectively. *n *= 5 mice per group.

We first mapped SV2B proteins in mouse CA3–CA1 synapses using immunogold staining and EM. As reported previously (Bajjalieh et al., 1994; Crèvecoeur et al., 2013, 2014), anti-SV2B antibodies labeled most areas of the forebrain, sparing some projections like the hippocampal mossy fiber pathway and the stratum lacunosum moleculare in CA1 (Figure 1A). In our EM analysis, we focused on the stratum radiatum in CA1, where SV2B and SV2A were found to be expressed consistently (Figure 1A; Supplementary Figure S1A). Pre-embedding immunogold staining was optimized to maintain the ultrastructure and yield individual, homogeneous, and silver-intensified immunogold particles (SIPs). In images of ultra-thin tissue sections (70 nm) of stained stratum radiatum tissue recorded by scanning transmission EM (STEM; Figure 1C), SIPs could be automatically detected (see Materials and methods). Presynaptic structures of asymmetric synapses showed a high density of SIPs, whereas SV2B knockout mice were almost completely devoid of SIP signals (Figure 1C and D). SIP densities in dendritic spines and mitochondria were very low in both SV2B wildtype and knockout mice, indicating unspecific labeling (Figure 1D).

### Differential intrasynaptic distribution of SV2B and SV2A visualized by pre-embedding staining

Next, we compared SV2B and SV2A SIPs in asymmetric glutamatergic synapses and typically identified 1–4 SIPs in each synaptic cross-section (Figure 1E–G). Immunogold staining could not reveal all antigens in the tissue, and thus the number of SIPs is lower than the actual number of SV2 in synaptic cross-sections (see Discussion).

For each SIP or synaptic vesicle, we measured the shortest distance to the AZ in SV2B- or SV2A-labeled sections (Figure 1H and I). If SV2 is assumed to be present on every vesicle throughout the synapse, then the distribution of distance to the AZ of SIPs should be equal to that of synaptic vesicles. This was observed for SV2A but not the case for SV2B (Figure 1H and I). SV2B was infrequently found close to the AZ, i.e. only ∼10% of SV2B SIPs were detected within 40 nm to the AZ, whereas ∼29% of vesicles were located within this distance (Figure 1H). Then, the average probability of a vesicle being labeled by SV2B or SV2A was calculated per distance bin (Figure 1J), accounting for the slightly lower labeling efficacy of anti-SV2A antibodies (Figure 1G). The probabilities of observing SV2B and SV2A were quite similar on vesicles >80 nm to the AZ but differed significantly on vesicles close to the AZ and the synaptic cleft (0.03 and 0.17 for SV2B and SV2A, respectively) (Figure 1J), suggesting the lack of SV2B SIPs near the AZ and the differential intrasynaptic distribution of SV2A and SV2B.

### Post-embedding staining confirms the differential intrasynaptic distribution of SV2B and SV2A

To confirm the above findings, we performed post-embedding immunogold staining against SV2B and SV2A. Per the protocol, the tissue is first embedded in resin, then ultra-thin sectioned, and stained with antibodies tagged with larger gold particles that are directly visible via EM (not requiing silver intensification). In this approach, the tissue is preserved via light chemical fixation followed by cryo-fixation to preserve antigenicity and then freeze-substituted and embedded in resin. Therefore, antibodies do not have to penetrate tissue but rather bind to antigens on the surface of the section. In agreement with the pre-embedding results, SV2B-labeled gold particles were rarely observed close to the AZ (Figure 1K and M), while SV2A-labeled gold particles accumulated near the AZ (Figure 1L and N).

### Three-dimensional intrasynaptic localization of SV2B

**Figure 2:**
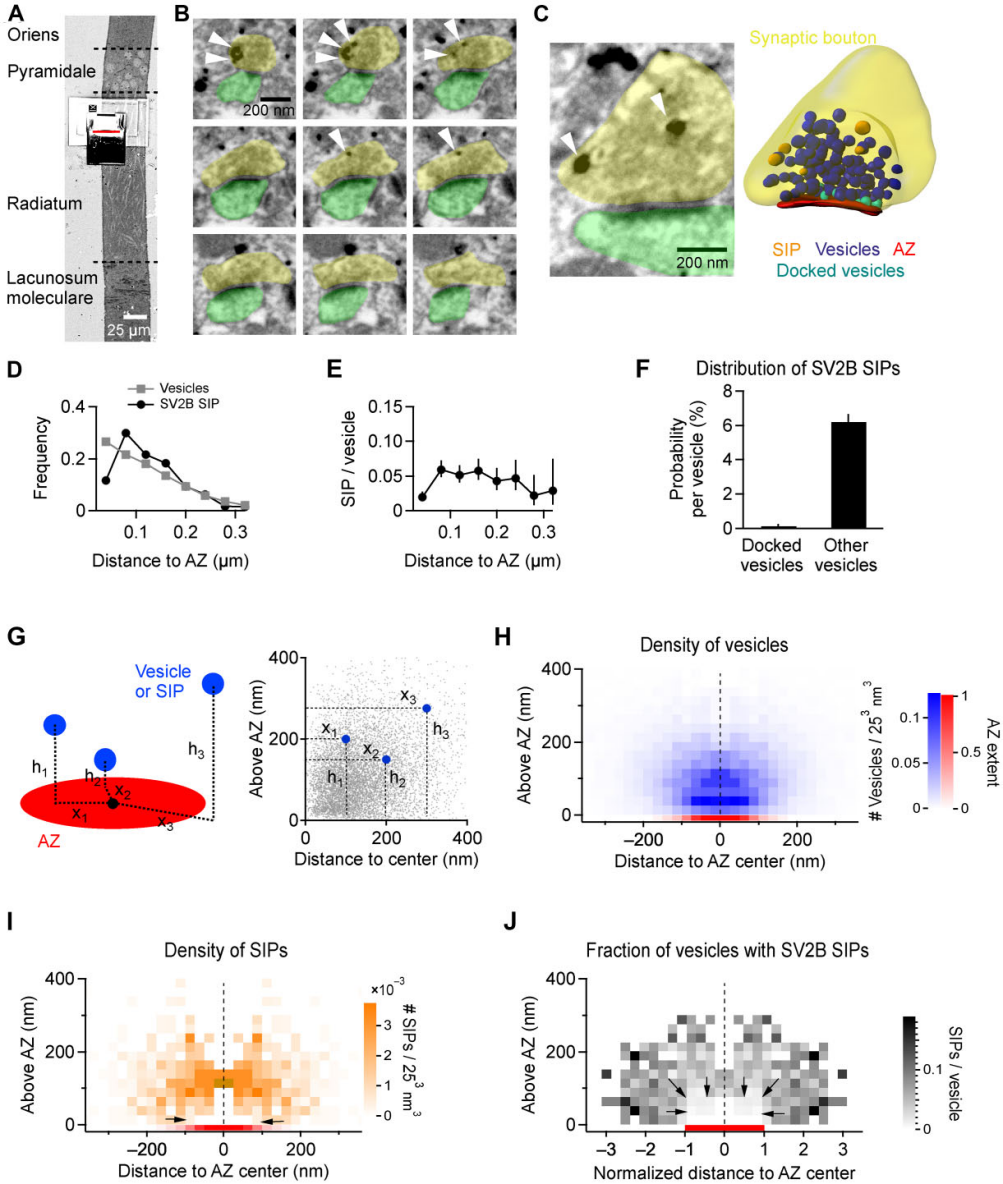
3D FIB-SEM imaging reveals that synaptic vesicles in the AZ domain are mostly devoid of SV2B. (**A**) Overview scan of back-scattered electrons after pre-embedding immunogold labeling of SV2B, showing different hippocampal layers in the hippocampal CA1 region and the region of interest for automated milling and imaging in FIB-SEM. The red line indicates the milling plane of the gallium beam. (**B**) A series of exemplary images from every fifth scan (25 nm) of a 3D FIB-SEM image stack. Color code: yellow, presynaptic bouton; green, spine. Arrowheads mark SV2B SIPs. (**C**) Exemplary image of a synapse after pre-embedding immunogold labeling of SV2B (left) and the corresponding 3D reconstructed synapse (right). Color code: yellow, presynaptic bouton; red, AZ; blue, synaptic vesicles; turquoise, docked synaptic vesicles; orange, SV2B SIPs. Arrowheads mark SV2B SIPs. (**D**) The frequencies of SV2B SIPs and synaptic vesicles relative to the distance from the AZ in 3D reconstructed synapses. SV2B-containing synaptic vesicles are mostly absent from the area proximal to the AZ and preferentially located at a distance of 100 nm from the AZ. *n *= 100 synapses. (**E**) The frequency of SV2B SIPs per synaptic vesicle along the AZ. The low ratio in the first bin close to the AZ is comparable to the 2D analysis result in Figure 1J. Data are shown as the ratio per bin ± 95% confidence interval of the ratio calculated across all synapses. *n *= 100 synapses. (**F**) The probability of SV2B labeling per synaptic vesicle is ∼50-fold higher for non-docked vesicles than for docked vesicles. *n *= 100 synapses. (**G**) The 3D coordinates of SIPs and synaptic vesicles relative to the center of the AZ are transformed into 2D coordinates by measuring the height above the AZ plane (*h*) and the distance of the foot of a dropped perpendicular to the center point (black dot) in the AZ plane (*x*). Color code: blue, example vesicles or SIPs; gray, all determined synaptic vesicles; red, AZ. (**H**–**J**) The 2D virtual cross-sections of the spatial densities of synaptic vesicles (**H**) and SV2B SIPs (**I**) and the fractions of synaptic vesicles with SV2B SIPs (**J**), revealing a clear spatial domain just above the AZ where vesicles very rarely carry SV2B. The brown bin shown in **I** represents the value of 6.4 × 10^−3^ SIPs/25^3^ nm^3^ and has not been included in the orange color scale. The cumulative distribution of the AZ extent is indicated in red at the bottom of the virtual cross-sections. For **H** and **I**, the bin size is 25^3^ nm^3^. For **J**, horizontal distances are normalized to the respective AZ radius, and the horizontal bin size represents an average equaling 25 nm. The AZ is always divided into four bins; due to the AZ normalization procedure, four smaller bins are generated for synapses with smaller AZs and *vice versa* for synapses with larger AZs. Data are mirrored at the vertical center line for clarity.

The exact position of a synaptic vesicle with respect to the AZ is considered an important predictor of whether and how rapidly the vesicle can be released (Park et al., 2012). The one-dimensional (1D) shortest distance analysis in two-dimensional (2D) electron micrographs as above provides a valid estimate, but it suffers from some inherent flaws caused by the section angle through a synapse. Additionally, the typical section thickness of 50–70 nm is well within the average size of synaptic vesicles, limiting the resolution and accuracy of synaptic vesicle mapping. Therefore, we aimed to perform a high-resolution three-dimensional (3D) analysis to correctly map SV2B-positive and SV2B-negative vesicles throughout synapses and improve the registration of their spatial association with the AZ. To this end, we combined pre-embedding staining with focused ion beam scanning EM (FIB-SEM). The ion beam is used for milling and exposing new surfaces of the tissue at 5-nm intervals, and the electron beam scans the structural image of the milled surface by acquiring back-scattered electrons. The milling window was placed in the stratum radiatum of CA1 (Figure 2A), and a series of typically 650 images (Figure 2B) were acquired for 3D analysis (Figure 2C). SIPs could be clearly identified across a depth of 30–59 nm (Figure 2B). We registered the position of each synaptic vesicle (a total of 6468) and each SIP (a total of 523) throughout 100 synapses and classified vesicles as docked (587) or non-docked (5881). Both the distributions of vesicles and SIPs to the AZ (Figure 2D) and SIP/vesicle ratios (Figure 2E) were in good agreement with those derived from the 2D image analysis, substantiating that synaptic vesicles close to the AZ are mainly devoid of SV2B. Moreover, in the first bin (0–40 nm) to the AZ, 6.2% ± 0.45% of the non-docked vesicles were labeled with SV2B, while only 0.13% ± 0.12% of the docked vesicles were labeled with SV2B (Figure 2F), suggesting that close to the AZ there is a steep decline of synaptic vesicles containing SV2B.

### A micro-zone at the AZ is almost devoid of SV2B-positive vesicles

We next used the full 3D coordinates of vesicles and SIPs to map their positions within the presynaptic bouton with respect to the center of the AZ. Based on a rendering of each AZ, we defined its center point (the black dot in Figure 2G) and the best leveling plane through that point (the red disc in Figure 2G). For each vesicle or SIP, we then measured its height above the AZ plane (*h*) and the distance from its foot of a dropped perpendicular to the center point of the AZ (*x*) (Figure 2G, left panel). Neglecting the azimuth angle, vesicles and SIPs can then be plotted as dots in a 2D graph (Figure 2G, right panel). Accordingly, we generated 2D histograms representing the spatial density of synaptic vesicles (Figure 2H) and SV2B SIPs (Figure 2I) above and beyond the AZ. Considering that the probability of observing either synaptic vesicles or SV2B SIPs increases with increasing *x* due to the fact that areas of more distant shells become progressively larger (size scales with *x*), these histograms represent 2D virtual cross-sections of an average synapse, and the value per bin (with a volume of 25^3^ nm^3^) represents the probability of a vesicle or a SIP located in that bin (at any azimuth angle). The AZ size varies across synapses, and the intensity of the red bar at the bottom indicates the relative frequency of observing a particular radius. Synaptic vesicles were primarily found above the AZ and accumulated near the AZ along its full width (Figure 2H). Notably, docked vesicles were found at almost equal spatial density over the AZ area, and thus the apparent radial gradient shown in Figure 1H is due to the variability of the AZ size (red bar; also see Supplementary Figure S1C for vesicle densities aligned to the AZ size). The overall intrasynaptic distribution of SV2B SIPs with respect to the AZ was similar to that of the vesicles, except that SV2B SIPs were rarely found in the first two lines of bins above the AZ (Figure 2I). We then generated the histogram illustrating the fractions of synaptic vesicles carrying SV2B depending on vesicle localization, where the parameter *x* within each synapse was normalized to the radius of the AZ within the same synapse to account for varying AZ sizes (Figure 2J). The histogram clearly shows a microdomain with sharp borders above the AZ up to a height of ∼75 nm but almost without vesicles carrying SV2B, further revealing that SV2B-carrying vesicles are indeed distributed in proximity to the presynaptic membrane, but only in regions outside the AZ (Figure 2J). As a result, clear and steep gradients appear on the sides and at the top of the AZ, which was not shown by 1D distance measurements, and ∼18% of the synaptic vesicles of each synapse were found in this microdomain almost completely devoid of SV2B SIPs (Figure 2J).

### Identification of non-vesicular SV2B residing at the cytoplasmic membrane of the peri-AZ

**Figure 3:**
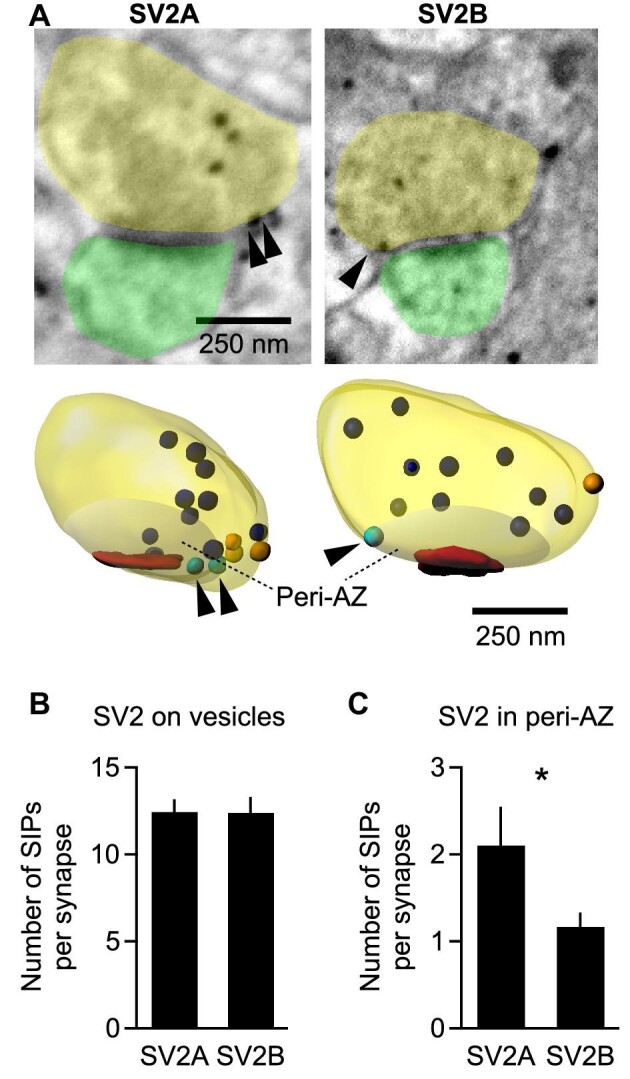
SV2B-labeled synaptic vesicles are found in the peri-AZ. (**A**) Exemplary images from 3D FIB-SEM stacks of pre-embedding immunogold-stained hippocampi showing SV2A-positive (left) and SV2B-positive (right) synapse and the corresponding 3D reconstructed synapses (lower panel). Arrowheads mark SIPs located on the membrane in the peri-AZ. Color code: yellow, presynaptic boutons; green, spines; red, AZ; blue, SIPs within the synapse; turquoise, SIPs on the membrane of the peri-AZ (shaded); orange, SIPs on the membrane outside the peri-AZ. The peri-AZ is defined as the membrane area surrounding the AZ with a diameter of 1.5-fold of the AZ radius (excluding the AZ itself). (**B**) The numbers of SV2A and SV2B SIPs on synaptic vesicles within each synapse are similar. (**C**) Significantly fewer SV2B SIPs than SV2A SIPs are found in the peri-AZ. For **B** and **C**, a total of 19 SV2A-positive synapses and 23 SV2B-positive synapses were analyzed, respectively.

The AZ is a part of the presynaptic membrane where synaptic vesicles are released most efficiently (Südhof, 2012) and docked vesicles are in a fusion-competent state. As SV2B is not contained in synaptic vesicles near the AZ, we wondered whether and how SV2B-positive vesicles can be released. While vesicle release cannot be directly captured in electron micrographs, the exocytosis process may generate detectable traces of release in these images. Following fusion with the membrane, the protein content of the synaptic vesicle membrane transiently becomes part of the presynaptic plasma membrane until it is re-used during an endocytic event (Soykan et al., 2016). Thus, during the cycle of exocytosis and endocytosis, electron micrographs may occasionally show vesicular proteins on the presynaptic cytoplasmic membrane. To count the number of SIPs on the presynaptic membrane in 3D reconstructed synapses, special care has to be taken to selectively count SIPs overlapping with the cell membrane and to exclude SIPs clearly belonging to docked vesicles or in proximity to the membrane. In this study, we excluded the AZ area, which is mainly devoid of SV2B, and counted SIPs on the cytoplasmic membrane of the peri-AZ, which is defined as the presynaptic membrane area with a diameter 1.5 times the AZ radius (Figure 3A). After we filtered synapses with strong labeling, ∼12 SIPs per synapse were found on both SV2A- and SV2B-labeled synaptic vesicles (Figure 3B). We found 2 ± 0.4 SV2A SIPs in the peri-AZ membrane area of each synapse and also found a few SV2B SIPs (1 ± 0.2 SV2B SIPs per synapse) in the peri-AZ (Figure 3C). This finding alone is not sufficient to prove exocytosis of SV2B-positive vesicles but suggests that SV2B-positive vesicles are likely to be released and recycled.

### A fraction of asymmetric synapses lack SV2B and are smaller than SV2B-positive synapses

**Figure 4:**
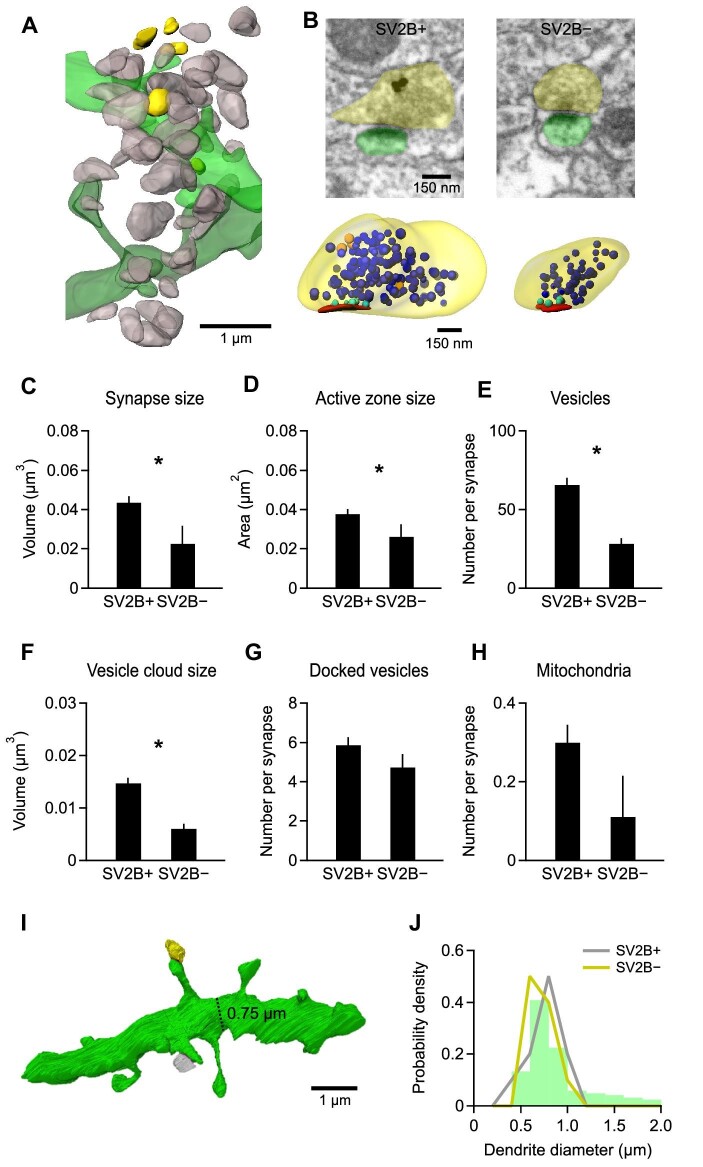
SV2B-positive and SV2B-negative synapses differ in size but connect to the same population of dendrites. (**A**) 3D reconstruction of SV2B-positive (gray) and SV2B-negative (yellow) presynaptic boutons as well as the connected spines and dendrites (green) from a FIB-SEM image stack. (**B**) Exemplary FIB-SEM images after pre-embedding immunolabeling of SV2B-positive (SV2B+) and SV2B-negative (SV2B−) synapses and the corresponding 3D reconstructed synapses (lower panel). Color code: yellow, presynaptic boutons; green, spines; red, AZ; blue, synaptic vesicles; turquoise, docked synaptic vesicles; orange, SV2B SIPs. (**C**–**H**) SV2B-negative synapses are significantly smaller than SV2B-positive synapses (**C**) and also exhibit a significantly smaller AZ area (**D**), fewer synaptic vesicles (**E**), and a smaller vesicle cloud size (**F**). The numbers of docked synaptic vesicles (**G**) and mitochondria (**H**) are slightly but not significantly lower (3D analysis). A total of 100 SV2B-positive synapses and 10 SV2B-negative synapses were analyzed, respectively. (**I**) 3D reconstruction of an exemplary dendrite (green) connecting with both an SV2B-positive (gray) and an SV2B-negative (yellow) synapse. The dendrite diameter is depicted by a dashed line. (**J**) Probability density distribution of all reconstructed dendrites with different diameters. SV2B-positive and SV2B-negative synapses are absent from dendrites with larger diameters. The diameter of every 10 dendrites connecting with SV2B-positive or negative synapses was measured.

We noted that 13% of the asymmetric synapses were completely devoid of SV2B SIPs (Figure 4A). Considering that 4.2 ± 0.3 SIPs per synapse were found via the 3D analysis (Supplementary Figure S1D), it is unlikely that SV2B in all these synapses were undetected due to low labeling efficacy. SV2B-negative synapses were ∼50% smaller than their SV2B-positive counterparts (Figure 4B) and showed proportionally smaller AZs as well as fewer vesicles and mitochondria, while the number of docked vesicles within each SV2B-positive synapse appeared comparable to that in a SV2B-negative synapse (Figure 4C–H). We then characterized the postsynaptic targets of SV2B-positive (*n *= 10) and SV2B-negative (*n *= 10) synapses by reconstructing the postsynaptic structure (Figure 4I). All synapses were found on spines of smaller-caliber dendrites (0.5–1 µm, Figure 4J) that likely represent higher-order (≥2nd) dendritic branches of CA1 pyramidal cells.

### Synaptic vesicles distant from the AZ are slightly more dispersed in SV2B knockout mice

To examine whether SV2B plays a role in organizing the synaptic ultrastructure or the position of the synaptic vesicle, we obtained FIB-SEM image stacks of Schaffer collateral synapses from SV2B wildtype and knockout mice (Supplementary Figure S2A). The number of synapses per tissue volume, synapse size, number of vesicles, and AZ area were unchanged in SV2B knockout mice compared with SV2B wildtype mice (Supplementary Figure S2B–H). However, the spatial distribution of synaptic vesicles within synapses slightly differed, i.e. more vesicles were found distal to the AZ in SV2B knockout mice (Supplementary Figure S2I), which was also revealed by 3D mapping of the vesicles (Supplementary Figure S2J), suggesting that the compactness of the cloud of vesicles is reduced in the absence of SV2B.

### Selective reduction of SYT-1 and SYT-2 expression levels in SV2B knockout mice

We next performed quantitative immunoblotting against SV2 family members as well as two subunits of the α-amino-3-hydroxy-5-methyl-4-isoxazolepropionic acid (AMPA) receptor with brain homogenates prepared from the cortex, hippocampus, and cerebellum of SV2B wildtype and knockout mice (Supplementary Figure S3 and Table S1). Similar to previously reported (Janz et al., 1999; Morgans et al., 2009), ablation of SV2B did not result in a compensatory upregulation of SV2A or SV2C or any changes in the protein levels of synapsin 1 (SYN-1), syntaxin-1 (STX-1), STX-7, VAMP-4, synaptogyrin 3, AMPA1, and GLUR2 (Supplementary Figure S3 and Table S1). However, both protein levels of synaptotagmin-1 (SYT-1) and SYT-2 in hippocampal lysates and the expression levels of SYT-1 in cortex and cerebellum homogenates were significantly reduced upon SV2B knockout (Supplementary Figure S3 and Table S1).

### SV2B knockout mice exhibit an increased seizure threshold in a model employing high-frequency stimulation

Finally, SV2B wildtype and knockout mice were subjected to several behavioral paradigms and seizure models to test for functional changes at the system level. SV2B knockout mice exhibited a significantly increased threshold for seizure induction in the maximal electroshock (MES) model (Barker-Haliski and White, 2020), a validated model for generalized tonic–clonic seizures employing a brief and high-frequency stimulation (50 Hz; Supplementary Figure S4A). In all other tests, including the 6 Hz psychomotor seizure model of partial epilepsy (Barton et al., 2001), the actimetry test, the Y-maze test, the fear-motivated passive-avoidance test, and the rotarod test, no differences were observed between SV2B wildtype and knockout mice (Supplementary Figure S4B–H).

## Discussion

Our observation that docked vesicles do not, or only minimally, contain SV2B is contrary to the results of a previous study (Boyken et al., 2013). Boyken et al. (2013) employed mass spectrometry to analyze purified free and docked (containing the release machinery and membrane) synaptic vesicles and reported very comparable levels of SV2B and SV2A between the two preparations, suggesting the presence of SV2B at the AZ. Using the approach by Boyken et al. (2013), the purified docked vesicle–plasma membrane complexes were shown to contain long stretches of membrane to which a large number of vesicles are attached (Morciano et al., 2005, 2009). These vesicles contained not only docked vesicles but also numerous SV2B-positive non-docked vesicles that remained attached to docked vesicles during the isolation procedure. This contamination reduces the specificity of the ‘docked vesicle’ fraction, which may explain the discrepancy between the two studies. Notably, we analyzed a very specific and regionally restricted population of glutamatergic synapses, whereas Boyken et al. (2013) used the whole rat brain. At present, it is unknown whether our findings in hippocampal synapses apply to synapses in other brain regions.

In contrast to SV2B, the distribution of SV2A largely follows the distribution of synaptic vesicles throughout the synapse. This raises the question of whether SV2B-positive vesicles also carry SV2A. Pre-embedding staining for both SV2A and SV2B labeled only ∼7% of the synaptic vesicles (Figure 1G), suggesting that only a small fraction of synaptic vesicles express SV2 glycoproteins. On the other hand, the efficacy of antibody staining strongly depends on the protocol employed. In particular, staining protocols used for EM need to preserve the ultrastructure through strong fixation and little permeabilization, which typically results in the labeling of only a small fraction of the antigens present in the tissue. Indeed, sacrificing this constraint led to a dramatically increased number of labeled vesicles (Supplementary Figure S1B). Similarly, previous work on cultured neurons, requiring less permeabilization for staining, demonstrated labeling of the vast majority of synaptic vesicles with SV2 antibodies (Tao-Cheng, 2006, 2007). Furthermore, biochemical analysis of synaptic vesicles and synapses revealed that SV2A and SV2B are among the most abundant synaptic vesicle proteins (Morciano et al., 2005; Takamori et al., 2006; Grønborg et al., 2010; Mutch et al., 2011; Wilhelm et al., 2014), and a study of the protein content of single vesicles demonstrated that SV2 shows a highly reproducible copy number per vesicle (Mutch et al., 2011). We therefore hypothesize (also see Takamori et al., 2006) that in the most likely scenario, all synaptic vesicles analyzed in our study contain SV2A and most also contain SV2B, except for ∼18% of the vesicles in the AZ domain, which almost lack SV2B and carry SV2A only. Based on calibrating the protein content of a bulk synaptosomal preparation, it was previously estimated that a single vesicle contains 2–13 copies of SV2 (Takamori et al., 2006; Wilhelm et al., 2014). Possibly the most precise estimate comes from quantitative immunocytochemistry employing pan-SV2 antibodies, where the SV2 content of individual vesicles was 5 copies with very little variability across thousands of vesicles (Mutch et al., 2011). Based on this study, we assume that (i) every synaptic vesicle in total contains 5 copies of SV2, (ii) vesicles in the distal domain contain roughly equal amounts of SV2A and SV2B (on average 2.5 copies of each), and (iii) vesicles in the AZ domain contain 5 copies of SV2A only. Our data show that SV2A and SV2B both follow the distribution of synaptic vesicles in the distal domain, while SV2A shows a significantly (∼1.5-fold) higher labeling frequency on vesicles in the AZ domain than in the distal domain (Figure 1J).

The presynaptic AZ is a specialized area of the presynaptic plasma membrane where fast action potential-triggered synaptic vesicle release primarily takes place (Südhof, 2012). This raises the question of whether SV2B-positive vesicles that are not found near the AZ will ever be released. The fact that SV2B is present on the peri-AZ synaptic plasma membrane serves as indirect evidence that SV2B-positive vesicles are released and recycled by endocytosis. This conclusion is supported by the following observations: (i) synaptic vesicles throughout the synapse are recruited for release, albeit less frequently if remote from the AZ (Park et al., 2012; Rey et al., 2015); and (ii) SV2B is release-competent as it can restore neurotransmitter release after deletion of SV2A (Nowack et al., 2010) and drives release in synapses where only SV2B exists (Morgans et al., 2009; Wan et al., 2010). Therefore, we assume that the SV2A and SV2B SIPs found on the synaptic cell membrane indicate recycling of both SV2A/SV2B double-positive vesicles and SV2A-positive vesicles. The ratio of the count of SIPs on the membrane over the count of SIPs within the synapse can be viewed as a proxy for the relative amount of SV2 on the synaptic membrane to the amount of SV2 on vesicles. Consequently, we found 1/6 and 1/12 of the intrasynaptic content of SV2A and SV2B, respectively, on the membrane of the peri-AZ. These fractions are well within the range of what has previously been estimated for the vesicular protein SYT-1 based on live cell labeling approaches (Wienisch and Klingauf, 2006).

The segregation of SV2B-positive and SV2B-negative vesicles in intrasynaptic microdomains strongly suggests at least three possible scenarios within synapses. (i) Synaptic vesicles move through a synapse (Park et al., 2012), but SV2B-positive and SV2B-negative vesicles are not intermixed. This means that precise sorting or routing mechanisms must exist to ensure that SV2B-positive vesicles are not too close to the AZ and must apply to both newly generated and endocytosed SV2B-positive vesicles that are routed to the remote domain but do not reach the AZ. (ii) Endocytosis of vesicular proteins stranded in the membrane after fusion must be specific and produce SV2A-positive and SV2A/SV2B double-positive vesicles in a non-random fashion. If stranded SV2 is randomly integrated into endocytosed vesicles, ∼86% of endocytosed vesicles would carry both SV2A and SV2B (Supplementary Figure S5). As endocytosis must on average match exocytosis, this would not be consistent with the idea that most vesicles released stem from the AZ, where they only contain SV2A. (iii) It is most likely that upon biogenesis of synaptic vesicles, two types of vesicles are produced. One contains only SV2A and routes to the AZ domain, while the other contains SV2A and SV2B and resides and recycles in the distal peri-AZ domain. Synaptic vesicles have been proposed to be turned over and replaced with new ones within 24–48 h (Truckenbrodt et al., 2018). A simple assumption is that the lifetime of a vesicle depends on its usage, i.e. the number of recycling rounds. Truckenbrodt et al. (2018) suggested that vesicles are replaced after ∼100 rounds of recycling. Then, reckoning that the release rate of SV2A-positive vesicles is 1–2 times that of SV2A/SV2B double-positive vesicles, up to two times more SV2A vesicles should be generated to fulfill the demand. If only one type of mixed SV2A/SV2B double-positive vesicles are produced initially, following a selective endocytosis process, SV2B would progressively accumulate on the membrane. There could be a specific degradation pathway to get rid of the membrane-stranded SV2B molecules, but it seems inefficient.

In synapses of retinal bipolar neurons expressing only SV2B but not SV2A, deleting SV2B produces a profound reduction in vesicle fusion, demonstrating that SV2B also functions to promote vesicle release (Wan et al., 2010). The situation is different from synapses normally expressing both SV2A and SV2B paralogues; removal of SV2A produces a robust reduction in transmitter release (Custer et al., 2006; Bradberry and Chapman, 2022), whereas transmitter release is only mildly affected upon removal of SV2B (Custer et al., 2006). The reason for this difference has so far remained unresolved. The subsynaptic segregation of SV2A and SV2B to different vesicles shown here may provide a novel explanation. In response to action potentials, vesicle release occurs primarily at the AZ. As vesicles in this zone are largely devoid of SV2B, deleting SV2B may not grossly alter single action potential-driven release from the AZ. On the contrary, SV2A is enriched in vesicles clustered in the AZ, such that its deletion can readily affect action potential-stimulated secretion. In this study, a number of behavioral assays did not reveal abnormalities at the system level in SV2B knockout mice, except for the MES seizure model, where the threshold required to elicit a seizure was increased in mice lacking SV2B (Supplementary Figure S4A). To induce seizures in this model, high-frequency stimulation (50 Hz) was required (Supplementary Figure S4B). This may indicate that SV2B facilitates release in response to longer-lasting or higher levels of activity, which either recruits SV2B into the AZ or transiently leads to a higher cytosolic calcium concentration, causing SV2B-positive vesicles to be released in the peri-AZ. While the synaptic ultrastructure looked overall comparable between SV2B knockout and wildtype mice, we found a slight increase in the number of synaptic vesicles in the remote domain of the synapse (Supplementary Figure S2), possibly a consequence of a reduced release of distal vesicles in the peri-AZ. However, it should be noted that our ultrastructural analysis was only performed in the hippocampus, which itself does not play a major role in the behavioral and seizure assays. We consider the hippocampal synapse to be a model synapse for small glutamatergic synaptic boutons and hypothesize that SV2B might also be absent from the AZ of small glutamatergic boutons in other brain regions. However, there are currently no data to validate this generalization.

In summary, we describe the spatial organization of SV2A and SV2B on synaptic vesicles and the synaptic membrane based on the reconstruction of entire synapses, which provides clear evidence that synaptic vesicles within a synapse can be molecularly heterogeneous, sorted robustly with high specificity, and maintained at steep gradients according to their protein equipment. As the AZ demarcates the borders of the domains and gradients of SV2A-positive and SV2A/SV2B double-positive vesicles, it is possible that the segregation of SV2 reflects the functional specialization of the two types of vesicles. It will be important to assess whether SV2A-positive vesicles correspond to vesicles in a ‘recycling pool’ and whether SV2A/SV2B double-positive vesicles correspond to a reserve pool of vesicles used for higher levels of activity and/or spontaneous release.

## Materials and methods

### Animals

All animal experiments were conducted according to the guidelines of the University of Bonn Medical Centre Animal Care Committee and the guidelines from the European Directive (2010/63/EU) for the protection of animals used for research purposes.

For this study, 5- to 8-week-old male C57Bl6/N mice and SV2B wildtype and knockout mice (Janz et al., 1999) bred from Charles River were used. Mice were housed in a controlled environment under a 12-h light (7 am–7 pm) and dark (7 pm–7 am) cycle at a controlled temperature (22°C ± 2°C) and humidity (55% ± 10%) with food and water ad libitum. Mice were perfused transcardially with 4% paraformaldehyde (PFA; Merck) for fluorescent immunohistochemical staining, with 4% PFA and 2.5% glutaraldehyde (GA; AppliChem) in 1× phosphate-buffered saline (PBS) for no-antibody EM staining, or with 4% PFA and 0.5% GA for pre-embedding and post-embedding EM.

### Immunohistochemical staining

Brain slices (50 µm) from SV2B wildtype and knockout mice were incubated with rabbit anti-SV2B antibody (1:500, Synaptic Systems 119102) and 0.06% Triton X-100 (Sigma-Aldrich) in Tris-buffered saline (TBS) overnight at 4°C. Then, the slices were incubated with biotinylated goat anti-rabbit secondary antibody (1:167, Vector Laboratories BA-1000) and 0.06% Triton X-100 in TBS for 3 h at 35°C. Thereafter, the slices were incubated with Streptavidin Alexa Fluor 488 (1:167, Invitrogen S32354) and 0.06% Triton X-100 in TBS for 3 h at 35°C. Images were obtained with an inverted fluorescence microscope Eclipse Ti (Nikon) equipped with a 10× Plan Fluor objective (Nikon), a DS Qi2 camera (Nikon), and the Nikon NIS-Elements AR 5.11.03 acquisition software.

### Pre-embedding immunogold labeling

Brain slices (50 µm) were blocked with 10% normal goat serum (NGS; Aurion) and then incubated with rabbit anti-SV2B (1:100) or rabbit anti-SV2A (1:50, Synaptic Systems 119002) antibody, 2% NGS, and 0.1% Tween 20 in TBS overnight at 4°C. After washing, the slices were blocked with 2% NGS, 1% bovine serum albumin (BSA; Aurion), and 0.1% cold-water fish skin (CWFS) gelatin (Aurion) in TBS. Thereafter, the slices were incubated with goat anti-rabbit ultrasmall immunogold-labeled secondary antibody (1:50, Aurion), 2% NGS, 0.1% BSA-c (Aurion), and 0.1% CWFS gelatin in TBS overnight at 4°C. Subsequently, the slices were washed and fixed in 2% GA in PBS for 1 h and then rinsed before immunogold intensification with the silver enhancement reagent (R-Gent SE-EM, Aurion). After washing, the slices were post-fixed in PBS supplemented with 0.5% OsO_4_, then incubated in 1% uranyl acetate (Science Services) in distilled water, washed in distilled water, and dehydrated with an ascending ethanol (VWR) series. Subsequently, ethanol was removed by incubation in propylene oxide followed by infiltration with Epon.

### Post-embedding immunogold labeling

Brain slices (100 µm) were cryo-protected in 10%, 20%, and 30% glycerol. Then, 1 mm^2^ areas of interest were cut out of the slices, frozen in isopentane, and further processed in the Leica EM AFS2. The samples were freeze-substituted in a solution containing 0.1% uranyl acetate in 96% acetone (VWR), 1% methanol, and 3% water and subsequently embedded in Lowicryl HM20 Monostep resin (Polyscience). Serial 50-nm-thick sections were collected on gold slot grids as above. The grids were incubated in TBS supplemented with 0.1% NaBH_4_ and 50 mM glycine (Roth) and then incubated with rabbit anti-SV2B (1:100) or rabbit anti-SV2A (1:100) antibody, 2% NGS, and 0.01% Triton X-100 in TBS overnight at 4°C. Subsequently, the grids were incubated with goat anti-rabbit 10-nm immunogold secondary antibody (1:20, Aurion), 2% NGS, 0.01% Triton X-100, and 0.5% polyethylene glycol (AppliChem) in TBS. Afterwards, the grids were counterstained with 2% uranyl acetate and 3% lead citrate.

### EM imaging

Brain slices (50 µm) were washed with 0.1 M cacodylate buffer (Roth) and then post-fixed with 1% OsO_4_ (Science Services) and 0.8% K-ferricyanide (Roth) in 0.1 M cacodylate buffer for 2 h. After rinsing in 0.1 M cacodylate buffer, the sections were dehydrated with an ascending ethanol series and stained with 0.5% uranyl acetate in 70% ethanol, followed by infiltration with Epon (Sigma-Aldrich) and embedding. Epon-embedded brain slices were trimmed and polished with a Leica EM UC6 ultramicrotome.

All electron microscopic images were obtained by FIB-SEM with Helios G4 CX (Thermo Fisher Scientific) or Crossbeam 550 (Zeiss). Ultra-thin sections (50–70 nm) were scanned with a retractable STEM detector at 30 kV and 43 pA with a pixel size of 1.2–4 nm. 3D image stacks were recorded by automatically alternating gallium beam-based milling (5–10 nm thickness) and image acquisition (back-scattered electrons, in-column detector; 2 kV and 0.34 nA) using the Auto Slice and View 4.1 (Thermo Fisher Scientific) or Atlas 5 (Zeiss) software (pixel size 3 nm × 3 nm).

### Immunoblotting

Briefly, protein lysates from mouse hippocampus and cortical and cerebellar tissues were run on SDS–PAGE gels and then immunoblotted as previously described (Müller et al., 2022). An extended protocol and detailed information about the antibodies used can be found in Supplementary material.

### Behavioral and seizure tests

All *in vivo* experiments were performed and analyzed by investigators who were blinded to the genotypes. Adult SV2B knockout male mice (8–20 weeks old) and their age-matched wildtype littermates were tested in each experiment. Experimental details about the behavioral (actimetry, rotarod, Y-maze spontaneous alternation, and passive avoidance) tests, as well as the psychomotor 6 Hz and MES seizure threshold tests, are described in Supplementary material.

### Data analysis

The EM data were analyzed with the EspINA software package (EspINA Interactive Neuron Analyzer; Morales et al., 2013), except that the SV2B SIPs per area were analyzed with the ImageJ macro golddigger (from Sjollema and Giepmans). Synapse volumes, AZ (defined as the area on the presynaptic membrane that was directly opposite to the postsynaptic density), SIPs, synaptic vesicles, and vesicle densities were manually rendered in EspINA. The number of mitochondria per synapse was counted manually. Synapse volumes and vesicle cloud volumes, defined by the outer hull curve of the synaptic vesicles, were calculated by using the ‘physical size’ parameter, and AZ areas were reported according to the ‘synaptic apposition surface’ given by EspINA (Morales et al., 2013). The shortest distances from synaptic vesicle or SIP surfaces to the AZ surface in 2D and 3D were computed by EspINA. To transform this value into distances from the surface of the AZ to the center point of each SIP or synaptic vesicle, we added the individual SIP or synaptic vesicle radius (20 nm for all) to the distances provided by EspINA. Synaptic vesicles were classified as ‘docked’ if their distance to the AZ was 20–23 nm. This definition of distance for docked vesicles was validated by manual counting and visual inspection of individual docked vesicles in 30 synapses.

The position of a vesicle in relation to the AZ was expressed in terms of the in-plane distance of its projection onto the AZ plane to the AZ center and its height above the AZ plane. These calculations were based on information obtained from the rendered synapses in EspINA. Namely, we used the EspINA parameters ‘Centroid X’, ‘Centroid Y’, and ‘Centroid Z’ as the center coordinates and the ‘Binary Principal Axes’ as the AZ eigenvectors, where the vector given by the values of the binary principal axes (0,0), (0,1), and (0,2) corresponded to the eigenvector with the smallest eigenvalue. It was used as the normal to the AZ plane, which was defined by the first two eigenvectors. The projection *p*′ of the vesicle center *p* onto the AZ plane was then calculated as described in the following equation, with *q* being the AZ center and *n* being the unit normal vector onto the AZ plane:


\begin{eqnarray*}
p^{\prime} = {\mathrm{\ }}p - {\mathrm{\ }}\frac{{\left( {p - q} \right) \times n}}{{n{\mathrm{\ }} \times n}}{\mathrm{\ }} \times n.
\end{eqnarray*}

The projection *p*′ was then used to calculate the distances *x* and *h* from the AZ center and from the vesicle center, respectively. For visualization, see Figure 2G.


\begin{eqnarray*}
x &=& \left|\left| {q - {\mathrm{\ }}p^{\prime}} \right|\right|,\\
h &=& \left| {\left| {p - p^{\prime}} \right|} \right|.
\end{eqnarray*}

Rendered 3D synapses were illustrated with Imaris 9.1.0. Postsynaptic contacts were semi-automatically rendered with Ilastik 1.3.2 and analyzed with Blender software 2.79 and the software tool NeuroMorph (Jorstad et al., 2018).

Statistical analysis was performed with Prism GraphPad (version 8.4.3). *P < 0.05 was considered statistically significant. Unless otherwise stated, the data are shown as mean ± standard error of the mean.

## Supplementary Material

mjad054_Supplemental_File
