# Supplementary material for: SV2B defines a subpopulation of synaptic vesicles
Source: J Mol Cell Biol. 2023 Sep 8;15(9):mjad054. doi: 10.1093/jmcb/mjad054 (PMC11184983; doi:10.1093/jmcb/mjad054)
Supplement: mjad054_Supplemental_File [file mjad054_supplemental_file.pdf]

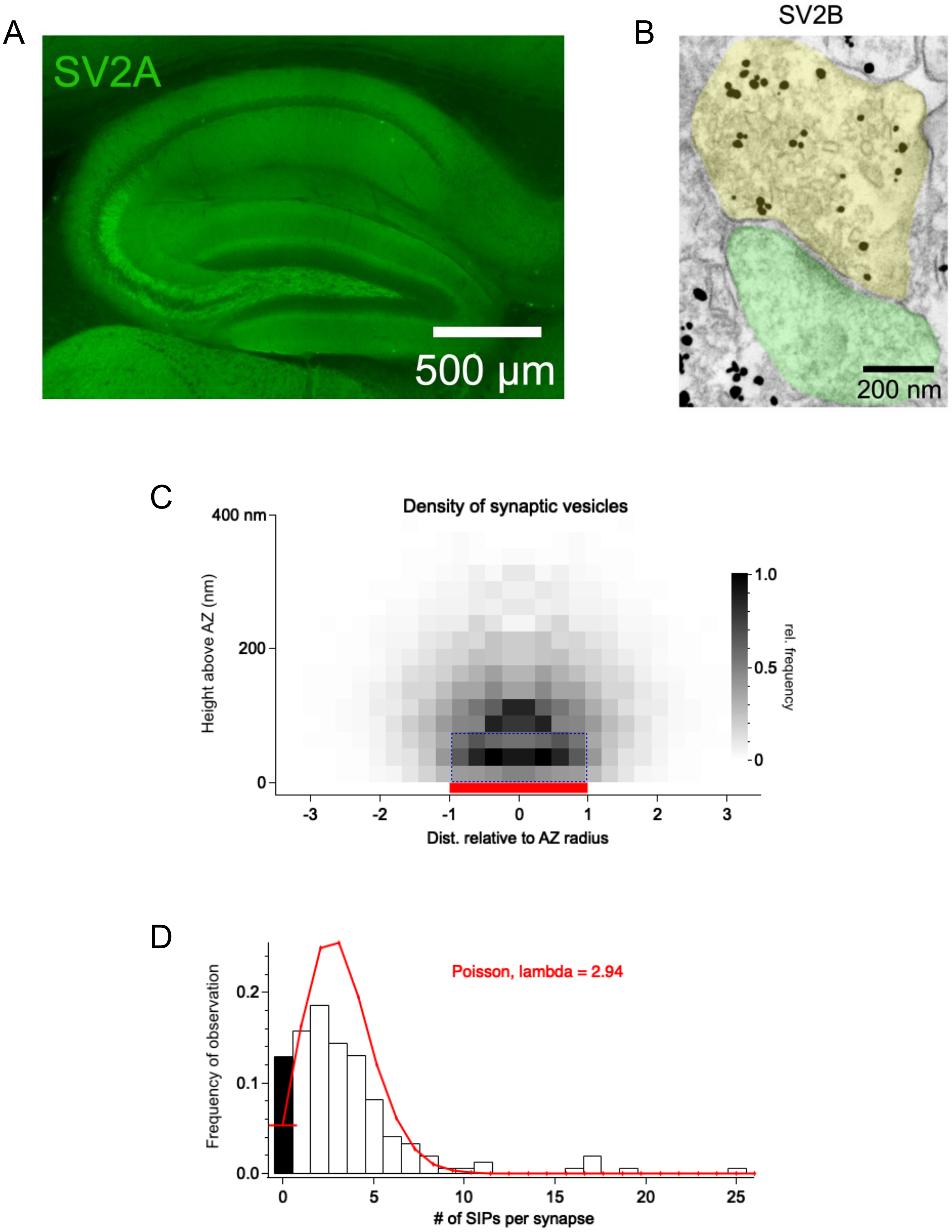

Supplementary Figure S1

Supplementary Figure S1: SV2A distribution, alternative SV2B pre-embedding staining, vesicle density with respect to AZ and distribution of SV2B SIP counts per synapse

(A) Exemplary image of a mouse hippocampus immunolabeled with anti-SV2A antibody. Note the robust staining in CA1 stratum radiatum.

(B) Example STEM image of a synapse in the stratum radiatum of CA1 showing silver-intensified immunogold particles (SIPs) after labeling with an anti-SV2B antibody. Many more SIPs per synapse can be achieved with harsher staining protocols. Color code: yellow = presynaptic bouton; green = spine.

(C) Virtual 2D cross section through an average synapse representing the spatial density of synaptic vesicles. In this panel all horizontal distances within each synapse (x) were divided by the AZ radius of the respective synapse. The relative AZ length is shown in red.

(D) Frequency of observations of SV2B SIPs per synapse (white bars). The red line indicates a fit of a Poisson function with lambda = 2.94 to the white bars. The black bar denotes the fraction of unlabeled synapses. Note that some unlabeled synapses are expected to occur solely due to random variability of the SIP count, indicated be the left most point/horizontal red bar of the Poisson line. However, we observed many more synapses without SIPs than predicted, suggesting there is a subpopulation of up to ~10% synapses not expressing SV2B.

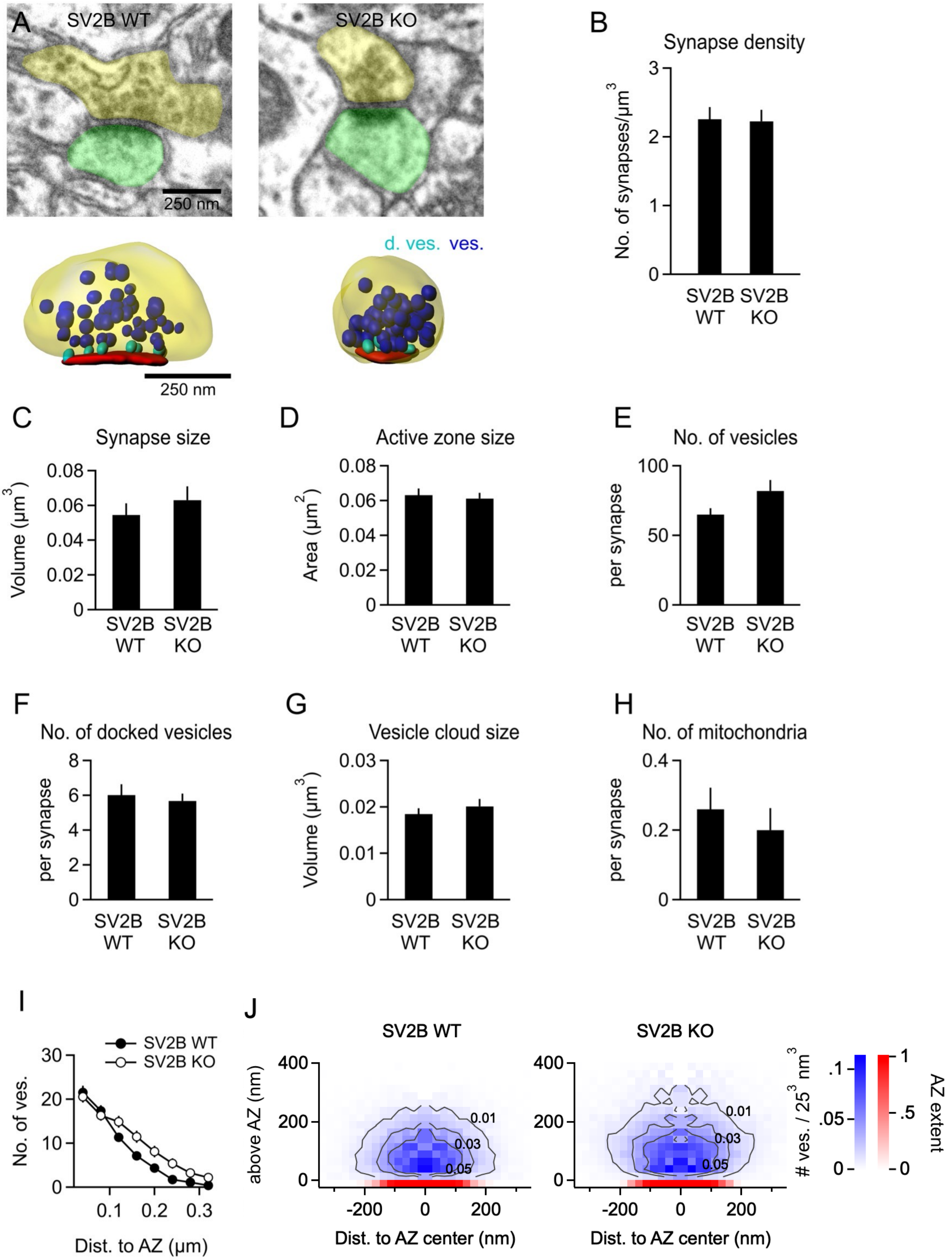

Supplementary Figure S2

Supplementary Figure S2: Unaltered synaptic ultrastructure in SV2B KO mice

(A) Example images from 3D FIB-SEM image stacks of SV2B WT (left) and SV2B KO (right) mouse brain sections (upper row) and of the corresponding 3D reconstructed synapses (lower row). Color code: yellow = presynaptic boutons; green = spines; red = AZs; blue = synaptic vesicles (ves.); turquoise = docked synaptic vesicles (d. ves.). Both scale bars: 250 nm.

(B) Synapse density of excitatory synapses in the stratum radiatum of CA1 does not differ between SV2B WT and KO mice. N=50 synapses for both SV2B WT and KO.

(C-H) Unaltered synapse size (C), AZ size (D), number of synaptic vesicles (E) and docked synaptic vesicles (F), vesicle cloud size (G) and number of mitochondria (H) in SV2B KO mice compared to SV2B WT mice (3D analysis). N=50 synapses for both SV2B WT and KO.

(I) The distribution of synaptic vesicles (ves.) relative to the AZ in SV2B WT and KO synapses was not significantly altered. N=50 synapses for both SV2B WT and KO.

(J) Virtual cross-sections through SV2B WT (left) and SV2B KO (right) synapses. Data are normalized on the AZ radius (red bar). Contour lines illustrate spatial vesicle densities. Color code: blue = synaptic vesicles; red = AZ. The synaptic vesicle cloud is slightly more compact in SV2B WT synapses and extends more distally to the AZ in KO mice.

(B-H) Data are shown as mean ± SEM. Unpaired t-test.

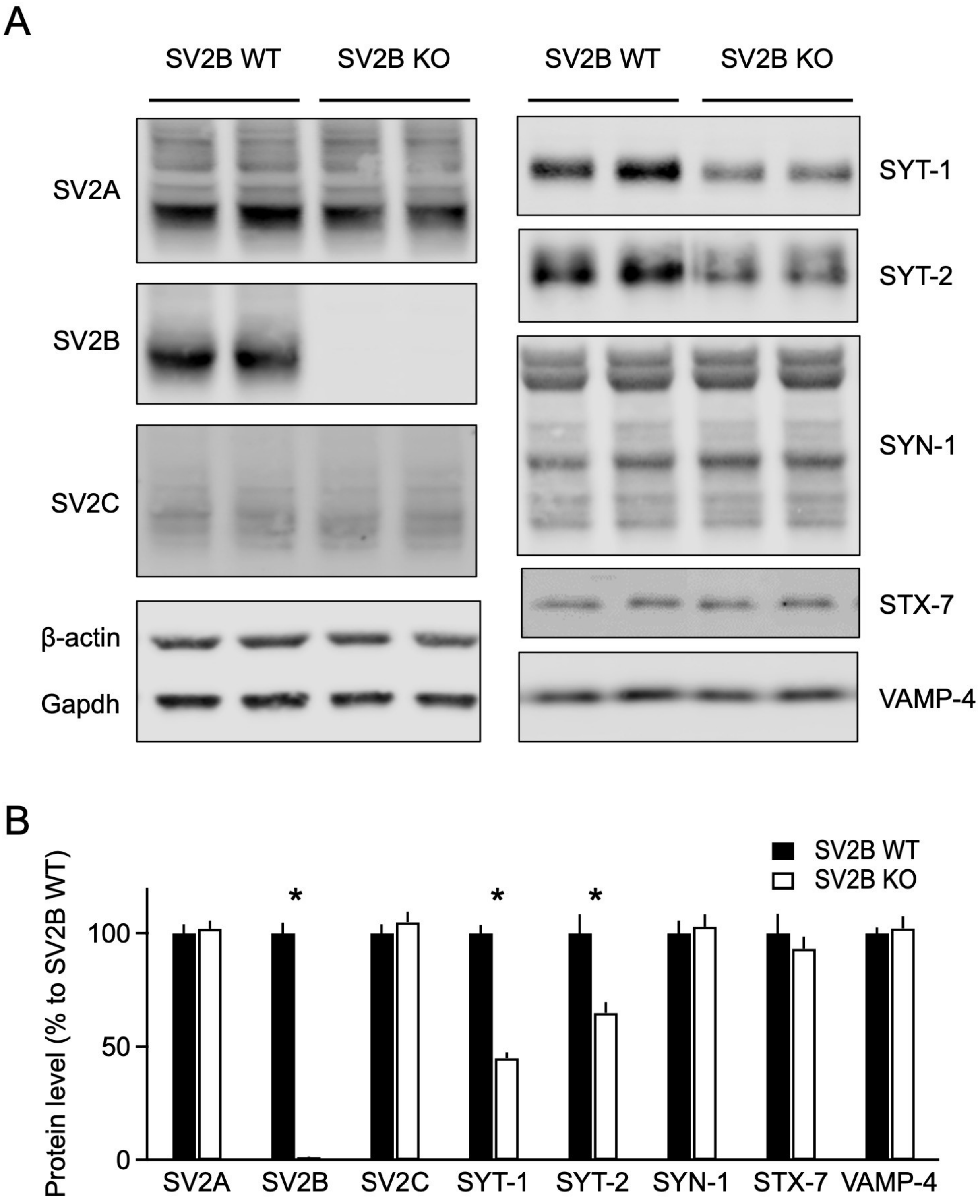

Supplementary Figure S3

Supplementary Figure S3: Synaptotagmin-1/2 levels are reduced in the absence of SV2B

(A) Representative immunoblots of SV2A (82 kDa), SV2B (77 kDa), SV2C (82 kDa), Synaptotagmin-1 and 2 (SYT-1 and SYT-2, respectively; 65 kDa), Synapsin-1 (SYN-1; 74 kDa), Syntaxin-7 (STX-7, 29 kDa), VAMP-4 (14 kDa), β-actin (41 kDa) and Gapdh (37 kDa) from hippocampus homogenates from SV2B WT and KO mice.

(B) Quantitative analysis of immunoblots shown in (A) reveals significantly reduced SV2B, Synaptotagmin-1 and -2 protein levels. N=10 animals for SV2A, SV2B, SV2C, SYT-1, SYN-1 SV2B WT and KO; n=8 animals for SYT-2, STX-7, VAMP-4 SV2B WT and n=6 animals for SYT-2, STX-7, VAMP-4 and SV2B KO. Note that the data does not allow to compare abundance across proteins. Data are shown as mean ± SEM. Statistical analysis was performed by Mann-Whitney U-test.

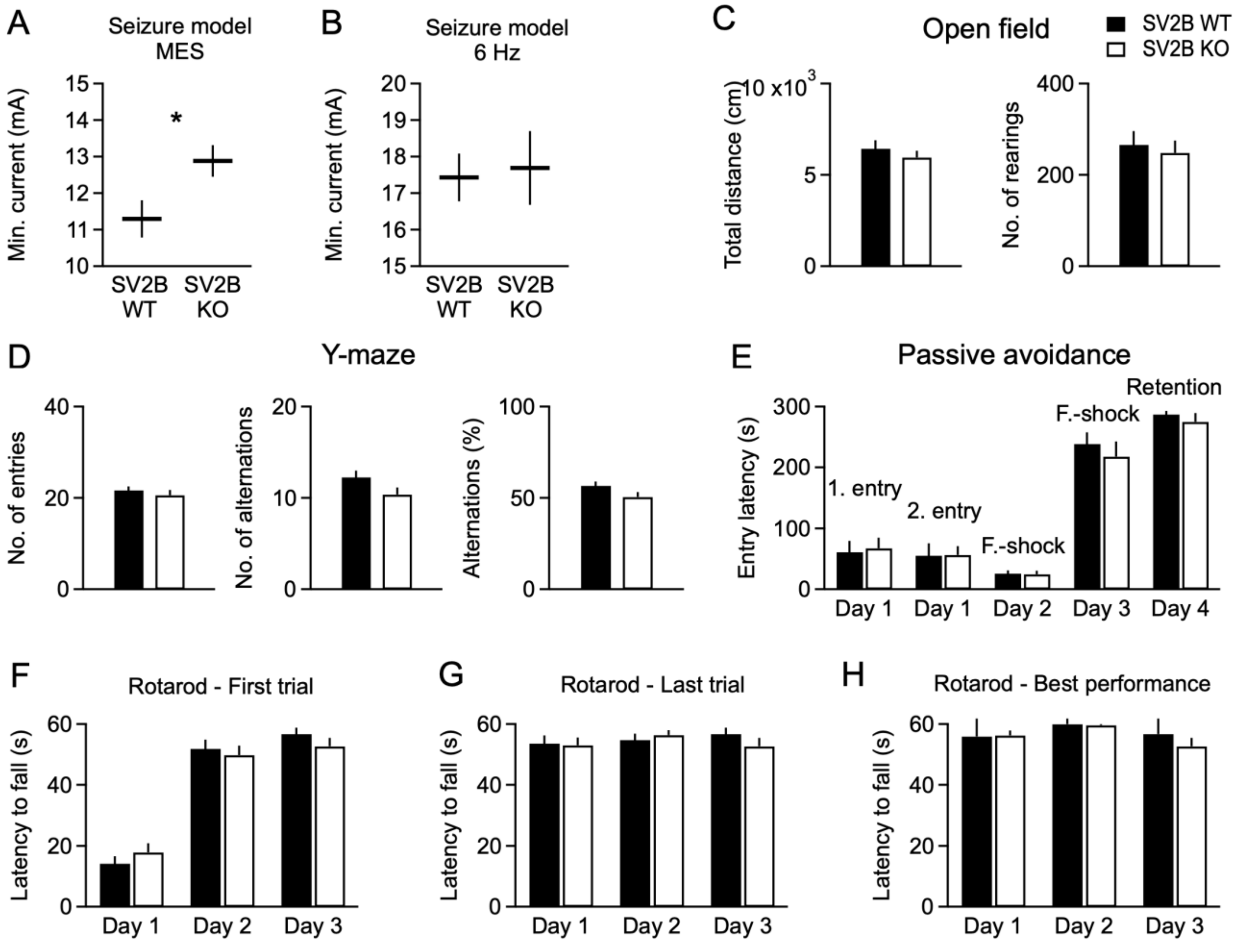

Supplementary Figure S4

Supplementary Figure S4: SV2B KO mice exhibit an increased seizure threshold in the maximal electroshock model but are inconspicuous in other behavioral tests

(A) Significantly increased average minimal current necessary to trigger tonic seizures in SV2B KO mice with the maximal electroshock (MES) model. N = 10 (SV2B WT) and 9 (SV2B KO) animals.

(B) Unaltered average minimal current necessary to trigger partial seizures in SV2B KO mice with the 6 Hz seizure model. N = 14 (SV2B WT) and 13 (SV2B KO) animals.

(C) SV2B KO mice behave in the Open field (actimetry) test like SV2B WT mice with same exploration distance (left) and number of rearings (right). N = 15 (SV2B WT) and 16 (SV2B KO) animals.

(D) SV2B KO mice behave in the Y-maze test like SV2B WT mice with same number of entries (left) and alternation probability (right). N = 15 (SV2B WT) and 16 (SV2 BKO) animals.

(E) Passive avoidance test measuring the latency to enter the chamber before and after a foot shock did not reveal any difference between SV2B WT and KO mice. N = 15 (SV2B WT) and 16 (SV2B KO) animals.

(F-H) Motor performance assessed by the rotarod on three consecutive days did not differ between SV2B WT and KO mice. (F) first and (G) last trial, (H) best performance of all trials. N = 15 (SV2B WT) and 16 (SV2B KO) animals.

(A-H) Data are shown as mean ± SEM.

(1) #A ~ RelRt_A + 0.5 RelRt_A/B

(2) #B ~ 0.5 RelRt_A/B

(3) RelRt_A/B = 2 RelRt_A

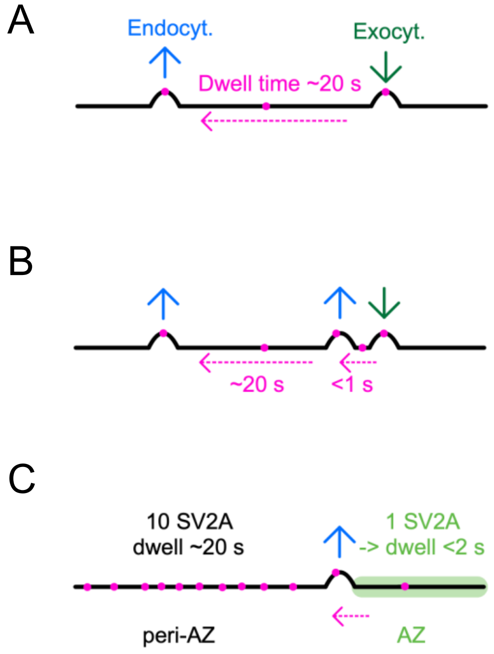

Supplementary Figure S5

Supplementary Figure S5: Estimating relative release and AZ clearance time of SV2s

Under the assumption that exocytosis and endocytosis are the only relevant modes of delivery and retrieval of SV2 proteins to and from the peri-AZ membrane, respectively, and that both SV2 paralogues show similar and constant dwell times in the cytoplasmic membrane (A), the number of SIPs in the peri-AZ membrane can yield information about the relative average release rates of SV2A/B+ and SV2A+ vesicles. As we render the synapse in its entity and therefore our counts reflect the underlying stoichiometry our data allows us to estimate this parameter. Every (full) exocytosis event will deliver its vesicular SV2 cargo to the membrane for the period of the dwell time (~20 s, (Soykan et al., 2016)). If the next vesicle is released during this dwell time – i. e. before the previously released material is endocytosed – material will accumulate in the membrane. In fact, the accumulation will increase proportionally to the release frequency – in analogy to the well-known single compartment model describing the rise in intracellular calcium as proportional to the action potential frequency (Helmchen et al., 1996). Our data lacks timing so we cannot determine release rates themselves, but, under our assumption made above, the SIP counts are still proportional to the average release rates and we can make a relative comparison between the synaptic release rates of SV2A+ and SV2A/B+ vesicles. In that simple model, the observed count of SV2A, #A, must be proportional to the sum of the release rate of SV2A+ and SV2A/B+ vesicles, RelRt_A, RelRt_A/B yielding (1). RelRt_A/B is multiplied by 0.5 as release of vesicles containing SV2A and B delivers only half of SV2A molecules compared to SV2A only containing vesicles. Similarly, we can write (2). As we know that there are twice more SV2A than SV2B molecules on the membrane we can in (2) substitute 0.5#A for #B, rearrange and substitute into (1) to obtain (3), i. e. we can derive a first rough estimate to predict that the total release rate of SV2A/B+ vesicles is twice that of vesicles containing only SV2A. This represents an upper estimate and needs some corrections for faster modes of endocytosis happening near the edge of the AZ, see below, but it already shows that release of SV2A/B+ vesicles cannot be a very rare phenomenon when compared to SV2A+ vesicles even though SV2A/B+ vesicles are not found near the AZ.

In response to low frequency firing, as it is typical for CA3 cells which provide the presynaptic terminals studied here, a fast, sub-second endocytosis component can be responsible for 1/2 if not 2/3 of vesicle recycling following action potential-triggered release from the AZ (Delvendahl et al., 2016; Soykan et al., 2017)see B). For this reason, the number of membrane SV2A SIPs may underestimate the release rate of SV2A in the calculation above by a factor of 2 to 3. Because we did neither observe SV2B near the AZ domain nor in the AZ membrane, we believe SV2B is not released at the AZ and may not undergo ultrafast endocytosis. Taking fast endocytosis into account, the average release rate of SV2A+ vesicle would be equal or ~50% higher than that of SV2A/B+ vesicles.

SV2A was consistently observed on docked vesicles but it was only rarely found in the membrane of the AZ. We counted on average only ~0.25 SV2A SIPs per synapse in the membrane of the AZ (data not shown), roughly 10-fold less than in the membrane of the peri-AZ (cf. Fig. 3). The likelihood to identify a SIP-labelled SV2 at a certain location in an EM image, such as the AZ, also depends on how much time it spends there. If SV2A rapidly moves through one location and rests at a different one, the likelihood it is found at the first and transient position is small (see C)). The fact that it is very likely that SV2A-containing vesicles are released at the AZ but we only rarely detect SV2A in the AZ membrane suggests that the protein is rapidly cleared from the AZ area. The 10-fold difference in SIP counts between AZ and peri-AZ membrane in turn indicates that SV2A stays at least 10-fold longer in the peri-AZ membrane than in the AZ. Assuming a dwell time in the peri-AZ before endocytosis of ~20 s (Soykan et al., 2016) our data would predict SV2A to not stay longer than ~2 s in the AZ membrane following fusion. The residency time could differ even more than 10-fold if some SV2A molecules would not end up in the peri-AZ and but were directly endocytosed at the edge of the AZ, implying that the time spent in the AZ membrane would be even shorter than 2 s. This is consistent with the view and findings that release sites are cleared within 1-2 s from synaptic vesicle material stranded in the membrane after fusion by lateral movement and ultrafast endocytosis at the edge of the AZ (Hosoi et al., 2009; Hua et al., 2011, 2013; Watanabe

et al., 2013; Gimber et al., 2015; Tehran and Maritzen, 2022). Thus, our counting of membrane associated SV2-labelling SIPs structurally supports the view that vesicular proteins are rapidly cleared from the AZ membrane at least 10-fold faster than they are endocytosed.

Further, this counting strongly suggests that upon endocytosis SV2A and B are assigned to new vesicles in a regulated and non-random manner. If stranded SV2 proteins would be randomly integrated into endocytosed vesicles, SV2A+ and SV2A/B+ vesicles would also be generated but the resulting fractions would not match the estimated release rates of SV2A+ and SV2A/B+ containing vesicles: 2/3 and 1/3 of the membrane pool of SV2 proteins are comprised of SV2A and B, respectively, and vesicles contain 5 copies of SV2 (Mutch et al., 2011). This would mean that endocytosis of synaptic vesicles carrying 5 SV2A molecules would happen with a probability of at most (2/3)^5 ~ 13% and 5 SV2B with a probability of at most (1/3)^5 ~ 0.4% and SV2A/B+ vesicles would occur in the remaining ~86% of cases. In other words, the random model would generate 6-7-fold more SV2A/B+ vesicles than SV2A+ vesicles which does not match the estimate that SV2A+ and SV2A/B+ are very likely released at similar rates if not SV2A+ vesicles are released more often (see above). To match those most probable release rates, endocytosis should be specific with regard to the equipment of endocytosed vesicles with SV2A and SV2B proteins. Indeed, mechanisms have been proposed potentially allowing for such preference like clustering of vesicular protein content post-fusion, keeping a pool of pre-assorted proteins next to the AZ ready for endocytosis, and a surface pool of stranded vesicles (Wienisch and Klingauf, 2006; Hua et al., 2011).

	Cortex	Hippocampus	Cerebellum
SV2A (n=10)	nc	nc	nc
SV2B (n=10)	KO	KO	KO
SV2C (n=10)	nc	nc	nc
Synaptotagmin 1 (n=10)	↓	↓	↓
Synaptotagmin 2 (n=8)	/	↓	/
VAMP4 (n=8)	/	nc	/
Synapsin 1 (n=10)	nc	nc	nc
Syntaxin 1 (n=10)	nc	/	/
Synaptogyrin 3 (n=10)	nc	/	/
GluA1 (n=8)	/	nc	nc
GluA2 (n=8)	/	nc	nc
Syntaxin 7 (n=8)	/	nc	/

Supplementary Table S1

Supplementary Table S1

Expression levels of the proteins listed in Table 1 were examined in lysates of cortex, hippocampus, and cerebellum from SV2B WT and KO mice by quantitative immunoblotting. Table 1 summarizes the observed results: nc = not changed between SV2B WT and KO; KO = SV2B knock-out, SV2B immunosignal absent; ↓ (downward facing arrow) = expression level of protein is decreased in the SV2B KO compared to WT; / = not determined. n indicates the number of mice analyzed per genotype (n = 8-10).

Supplementary Methods

Immunoblotting

Protein lysates were prepared from mouse hippocampus, cortical and cerebellar tissues using RIPA buffer (Sigma-Aldrich) containing protease and phosphatase inhibitors (Pierce). Tissue lysates were centrifuged at 10,000 rpm for 10 min at 4°C and supernatants were used for BCA measurements of protein concentration (BCA protein assay kit, Thermo Fisher Scientific, 23225, USA). Samples were prepared for SDS-PAGE in 1× Bolt LDS sample buffer (Thermo Fisher Scientific) and 1× Bolt antioxidant (Thermo Fisher Scientific), then heated for 5 min at 95°C. 10 μg of proteins were resolved on a 4–12% Bolt SDS-PAGE gel (Thermo Fisher Scientific) in MOPS running buffer (Thermo Fisher Scientific), then transferred to a PVDF membrane (Millipore). The membrane was blocked for 1 h in TBS Odyssey blocking buffer (Licor), followed by an overnight incubation with SV2A (1:4,000, Synaptic Systems 119002), SV2B (1:4,000, Synaptic Systems 119102), Synaptotagmin-1 (1:4,000, Synaptic Systems 105102), Synaptotagmin-2 (1:4,000, Synaptic Systems 105123), VAMP-4 (1:4,000, Synaptic Systems PA1-768), SV2C (1:4,000, Invitrogen PA5-59290), Synapsin1 (1:4,000, Cell Signaling Technology 5297), β-actin (1:7,500, Cell signaling Technology 3700S, 4970I), and Gapdh (1:7,500, Cell signaling Technology 97166, 5174) antibodies. Secondary antibodies (Thermo Fisher Scientific) were diluted 1:5,000. All washes were performed using TBS plus 0.1% Tween 20. Images were acquired by scanning with a Licor CLX and quantified using Image Studio software.

Behavioral and seizure tests

All in vivo experiments were performed and analyzed blind to the genotypes. Adult SV2B KO male mice (8-20 weeks old) and their age-matched WT littermates were tested in each experiment. Before seizure and behavioral tests, mice were habituated to the testing room for at least 30-60 min. Mice were housed in individual ventilated cages in the same air-conditioned room (temperature 22 ± 2°C, humidity 55 ± 15%, day/night cycle 12 h/12 h, light on from 6 am to 6 pm). Animals had free access to food and water. All in vivo experiments were conducted

in compliance with guidelines issued by the ethics committee for animal experimentation according to Belgian law. The experiments were performed in accordance with the European Committee Council directive (2010/63/EU). All efforts were made to minimize animal suffering.

Actimetry

A mouse was placed in the center of a clear, open chamber 22 x 21.5 x 40 cm from an infrared actometer system (Bioseb) and allowed to freely explore for 60 min. In each chamber, two layers of light beams (16 for each layer) in the horizontal X and Y directions capture the locomotor activity of the mouse. The horizontal travel and vertical activity were quantified using an Actitrack software (Bioseb).

Rotarod test

A mouse was placed on a rotarod apparatus (Ugo-Basile) set at a constant speed of 6 revolution per minutes. Each trial lasted for a maximum of 1 min. The latency for the mouse to fall from the rod was recorded for each trial. Mice were tested in three trials per day for 2 consecutive days and one trial during the third day. There was a 60 min resting interval between trials.

Y maze spontaneous alternation test

A mouse was placed at the extremity of a Y-shaped maze consisting of three walled arms 41 x 3 x 12.5 cm and allowed to freely explore the different arms for 8 min. The sequence of the arms that the mouse entered was monitored using a video camera placed above the maze. The correct choice refers to when the mouse entered an alternate arm after it came out of one arm.

Passive avoidance test

For step-through passive avoidance test, the experiments were performed in an automatically operated commercial passive avoidance apparatus (Ugo-Basile). The passive avoidance step-through cage was divided into two equal size compartments: light (white and illuminated) and dark (black and dark). The two compartments were separated by a partition which embodies an automatically operated sliding door at the floor level. During the first day of training trial, each mouse was initially placed in the light compartment, facing away from the dark compartment

(door closed and shock disconnected). After 15 s, the door automatically open and the latency to first entry into the dark compartment was recorded. After 15 s, the mouse was removed from the dark compartment and returned to its home cage. To accelerate the training phase, a second passage was allowed on the same day 3 h later by following the same procedure. On the second day (acquisition trial), a 0.3 mA mild foot-shock was delivered for 3 s when the mouse passed from the light into the dark compartment with all four paws in and the automated slide door was closed. After 15 s, the mouse was removed from the dark compartment and returned to its home cage. The latency to transfer into the dark compartment was measured during a maximum of 300 s (maximum cut off time allowed by the used apparatus). On the third day, another acquisition trial was performed by following the same procedure as during the second day. On the fourth day (retention trial), the latency to transfer from the light into the dark compartment was measured by following the similar procedure but without delivering the foot-shock.

Psychomotor 6 Hz seizure threshold test

Mice were stimulated (0.2 ms duration monopolar rectangular pulses at 6 Hz for 3 s) through corneal electrodes connected to a stimulator (ECT Unit 57800, Ugo-Basile, Comerio, Italy). A drop of Unicaïne (0.4% oxybuprocainum in saline) was placed on the eyes before the stimulation to induce local anesthesia and ensure good conductivity. During the stimulation, each mouse was manually restrained and then gently released into an observation cage (38 x 26 x 14 cm) immediately after the current application. The seizures were often preceded by a brief period (~2–3 s) of locomotor agitation (running and jumping). The animals then exhibited immobility associated with rearing, automatisms, forelimb clonus, twitching of the vibrissae, and, sometimes, Straub tail. The animals were observed for 30 s following the electrical stimulation. The main seizure endpoint was the duration of immobility. Mice resuming a normal behavior within 7 s after the end of the stimulation were considered as not displaying the seizure behavior. The mice were subjected to stimuli with different current intensities (11-26 mA) according to the "up-and-down" method (Giardina and Gasior, 2009). Each mouse was stimulated only once at any given current intensity and convulsant activity was judged as

described above. If the mice responded with seizures, the next mouse was stimulated with a current of an intensity 0.06-log step lower than the previous one. If the mouse did not exhibit seizures, the next one was stimulated with a current of an intensity 0.06-log step higher than the previous one.

Maximal electroshock seizure threshold test

Generalized tonic-clonic convulsions were induced by applying a sinusoidal alternating current (maximal output voltage 500 V, 50 Hz for 0.2 s) via corneal electrodes from a rodent shocker (WITT Industrie Elektronik, Berlin, Germany). A drop of Unicaïne (0.4% oxybuprocainum in saline) was placed on the eyes before the stimulation to induce local anesthesia and ensure good conductivity. During the stimulation, each mouse was manually restrained and then gently released into an observation cage (38 x 26 x 14 cm) immediately after the current application. Tonic hind limb extension (tonus; i.e., the hind limbs of animals out-stretched 180° to the plane of the body axis), which typically occurred within less than 10 s post stimulation, was taken as the behavioral endpoint reflecting seizure activity. Animals were observed for 5 min following the electrical stimulation. The mice were subjected to stimuli with different current intensities (8-14 mA) according to the "up-and-down" method (Giardina and Gasior, 2009). If the mouse had a tonic seizure with hindlimb extension, the next mouse was administered an electroshock at a 0.06-log step lower than the previous one. If the mouse did not exhibit tonic, the next mouse was administered an electroshock at a 0.06-log step higher intensity, the next one was stimulated with a current of an intensity 0.06-log step higher than the previous one.

References

Delvendahl, I., Vyleta, N. P., von Gersdorff, H. et al. (2016). Fast, Temperature-Sensitive and Clathrin-Independent Endocytosis at Central Synapses. Neuron 90, 492–498.

Giardina, W. J. and Gasior, M. (2009). Acute Seizure Tests in Epilepsy Research: Electroshock- and Chemical-Induced Convulsions in the Mouse. Curr Protoc Pharmacol 45, Unit 5.22.

Gimber, N., Tadeus, G., Maritzen, T. et al. (2015). Diffusional spread and confinement of newly exocytosed synaptic vesicle proteins. Nat Commun 6, 8392.

Helmchen, F., Imoto, K. and Sakmann, B. (1996). Ca2+ buffering and action potential-evoked Ca2+ signaling in dendrites of pyramidal neurons. Biophys J 70, 1069–1081.

Hosoi, N., Holt, M. and Sakaba, T. (2009). Calcium Dependence of Exo- and Endocytotic Coupling at a Glutamatergic Synapse. Neuron 63, 216–229.

Hua, Y., Sinha, R., Thiel, C. S. et al. (2011). A readily retrievable pool of synaptic vesicles.

Hua, Y., Woehler, A., Kahms, M. et al. (2013). Blocking Endocytosis Enhances Short-Term Synaptic Depression under Conditions of Normal Availability of Vesicles. Neuron 80, 343–349.

Mutch, S. A., Kensel-Hammes, P., Gadd, J. C. et al. (2011). Protein Quantification at the Single Vesicle Level Reveals That a Subset of Synaptic Vesicle Proteins Are Trafficked with High Precision. J Neurosci 31, 1461–1470.

Soykan, T., Kaempf, N., Sakaba, T. et al. (2017). Synaptic Vesicle Endocytosis Occurs on Multiple Timescales and Is Mediated by Formin-Dependent Actin Assembly. Neuron 93, 854-866.e4.

Soykan, T., Maritzen, T. and Haucke, V. (2016). Modes and mechanisms of synaptic vesicle recycling. Curr Opin Neurobiol 39, 17–23.

Tehran, D. A. and Maritzen, T. (2022). Endocytic proteins: An expanding repertoire of presynaptic functions. Curr Opin Neurobiol 73, 102519.

Watanabe, S., Rost, B. R., Camacho-Pérez, M. et al. (2013). Ultrafast endocytosis at mouse hippocampal synapses. Nature 504, 242–247.

Wienisch, M. and Klingauf, J. (2006). Vesicular proteins exocytosed and subsequently retrieved by compensatory endocytosis are nonidentical. Nat Neurosci 9, 1019–1027.
